# Novel Linear B Cell Epitopes of ASFV p54: Screening and Fine-Scale Mapping

**DOI:** 10.3390/microorganisms14071404

**Published:** 2026-06-25

**Authors:** Haili Wang, Wenying Yan, Xiao Liu, Yanwei Wang, Shulei Li, Linyi Bai, Xiaomin Li, Yaxin Guo, Aiping Wang

**Affiliations:** 1 Longhu Laboratory of Advanced Immunology, Zhengzhou 450046, Chinawenyingyan0909@163.com (W.Y.);; 2 Henan Provincial Key Laboratory of Immunological Biology, Zhengzhou 450001, China

**Keywords:** African swine fever virus, monoclonal antibodies, p54, B cell epitope, precise mapping

## Abstract

African swine fever (ASF) is an acute, febrile, and lethal pig disease induced by the African swine fever virus (ASFV). In the absence of an effective vaccine, early diagnosis is essential for the prevention and control of ASF disease. The p54 protein is important for ASFV diagnosis and vaccine design. In this study, ASFV p54 protein was constructed, expressed, purified, and used to generate three mAbs, namely 9A3, 5H2, and 2G6. Epitope mapping was performed using alanine mutants; the minimal linear epitope recognized by 9A3 and 5H2 was ^56^KKKAAAI^62^, and the minimal linear epitope recognized by 2G6 was ^108^TNRPATN^114^. Of these, ^56^KKKAAAI^62^ was identified as a new linear epitope for the first time. The epitopes were highly conserved in at least genotypes I and II. Alanine-scanning mutagenesis further revealed that residues 56K, 57K, 60A, 61A, 62L, 108T, 110R, 111P, 113T, and 114N were the core sites involved in antibody recognition. Overall, the mAbs and epitopes of the p54 protein identified in this study provide theoretical support for the development of ASFV vaccines based on the B cell epitope, the development of ASFV therapeutic antibody drugs, and the development of ASFV diagnostic tools.

## 1. Introduction

African swine fever (ASF) is an acute, contagious hemorrhagic disease caused by the African swine fever virus (ASFV). It affects domestic pigs and wild boar with a case fatality rate approaching 100% [1]. Since its first report in Kenya in 1921, ASF has spread globally in multiple waves (e.g., the first transboundary spread in 1957–1960 and a new round of cross-border spread since 2007), causing enormous economic losses to the swine industry. ASFV is a large double-stranded DNA virus encoding more than 150 proteins [2]. The virion has a complex structure and is highly stable in the environment. To date, no safe and effective commercial vaccine is available [3]. In the absence of effective vaccines, early and accurate diagnosis remains a critical measure for ASF prevention and control [4].

The ASFV p54 protein (encoded by the E183L gene) is an early-expressed structural transmembrane protein with a molecular weight of approximately 19.9 kDa [5]. The notable structural features of the p54 protein include a transmembrane domain, a Gly-Gly-X amino acid motif, and processing recognition sequences for various ASFV structural proteins, which determine its critical role in viral assembly and infection [6]. The p54 protein participates in the process of ASFV entry into host cells. Studies have reported that antibodies against p54 can inhibit viral adsorption to Vero cells adapted to ASFV strain BA71V [7]. However, the relevance of this finding to natural hosts (pigs and wild boars) remains unclear, as Vero cells are derived from the African green monkey kidney and are not a natural target cell type for ASFV. Moreover, the role of neutralizing antibodies in ASF is controversial. ASFV primarily infects macrophages, professional phagocytes that internalize large virions (>200 nm), mainly through phagocytosis rather than classical receptor-mediated endocytosis; in this context, antibody binding could potentially enhance infection via antibody-dependent enhancement (ADE), where opsonized virions are more efficiently phagocytosed and processed. Therefore, the neutralizing capacity of anti-p54 antibodies in natural hosts requires further investigation. ASFV p54 manipulates the secretory carrier membrane protein 3-dependent apoptotic bodies pathway to enhance cell–cell transmission [8].

The p54 protein is highly immunogenic and induces neutralizing antibodies following viral infection. It is a popular candidate antigen for the development of ASFV subunit vaccines and recombinant vaccines. Conjugation of the p54 protein with ferritin or encapsulin nanoparticles significantly enhances its immunogenicity, inducing durable immune activation and neutralizing antibody production [9]. DNA vaccines encapsulating p54 as a model antigen in lipid nanoparticles have been shown to induce high-titer-specific antibodies and T cell responses in pigs [10]. Expression of the p54 protein in the lapinized attenuated classical swine fever virus vaccine or recombinant adeno-associated virus has also yielded bivalent or multivalent vaccine candidates with good immunogenicity. The p54 protein is widely used as a target antigen in the serological detection of ASFV infection [11]. Studies have demonstrated that ELISA methods developed based on conserved linear epitopes of p54 exhibit high sensitivity and specificity, even outperforming some commercial kits [12]. Furthermore, lateral flow test strips developed using p54 as the core antigen provide new tools for the rapid, quantitative, point-of-care detection of ASFV antibodies.

Despite these promising applications, the linear B cell epitope landscape of p54 remains incompletely defined. Most previous studies have focused on limited regions, lacking a systematic, high-resolution scan of the entire p54 protein, which may have missed novel immunogenic sites. Based on the above background, this study aimed to comprehensively screen and finely map linear B cell epitopes on the ASFV p54 protein. We expressed and purified recombinant p54 protein, generated monoclonal antibodies, and systematically identified novel linear B cell epitopes using bioinformatics prediction, overlapping peptide scanning, and alanine-scanning mutagenesis. The key amino acid residues essential for antibody binding were determined. The novelty of this study lies in the following: (a) the first identification and precise mapping of the novel linear epitope ^56^KKKAAAI^62^; (b) the determination of core amino acids involved in antibody recognition for two epitope regions using alanine-scanning mutagenesis; (c) and the systematic analysis of the conservation of the identified epitopes across different ASFV genotypes (51 strains covering 22 genotypes). These results enrich the antigenic epitope map of the ASFV p54 protein and provide a theoretical basis for developing epitope-based diagnostic methods and multi-epitope vaccines against ASFV.

## 2. Materials and Methods

### 2.1. Cell Lines, Viruses, Reagents, and Serum

HEK293T cells were procured from ATCC (Manassas, VA, USA). Dulbecco’s modified Eagle’s medium was supplied by Gibco BRL, Paisley, UK. DMEM medium was purchased at Solarbio, Beijing, China. The plasmids of pET-28a, *Escherichia coli* (*E. coli*) DH5 α and Rosetta (DE3) were were purchased from Novagen (Darmstadt, Germany). Fetal bovine serum FBS was provided by Gibco-BRL (Middleton, WI, USA). Anti-ASFV p54 protein mAb was prepared in our laboratory. The synthetic peptide was synthesized by Sangon Biotech Co., Ltd. (Shanghai, China). Goat anti-mouse IgG-HRP, rabbit anti-pig IgG-HRP, and color developing solution TMB were purchased from Thermo Fisher Scientific ((Waltham, MA, USA)). The plasmid miniprep kit was a product of Novozymes (Nanjing, China). jetPRIME^®^ transfection reagent (catalog no. 114-01), Ni Sepharose^TM^ Excel resin (catalog no. 17-3712-02), and PEG 1500 (catalog no. 10783641001) were purchased from Polyplus-transfection^®^ (Illkirch-Graffenstaden, France); NcoI-HF and BamHI-HF restriction enzymes were purchased from New England Biolabs (Ipswich, MA, USA). T4 DNA Ligase and Premix Ex Taq^®^ DNA Polymerase were purchased from Takara Biomedical Technology Corporation, Ltd. (Beijing, China). HRP-conjugated goat anti-mouse antibody, HRP-conjugated goat anti-swine antibody, and Dylight 594-labeled goat anti-mouse IgG were purchased from Abbkine Scientific Corporation, Ltd. (Wuhan, China). eECL Western Blot Kit was purchased from CWbiotech Corporation, Ltd. (Taizhou, China). Mouse mAb subtype identification kit was purchased from Proteintech Corporation, Ltd. (Wuhan, China). IPTG, Ampicillin, BCA Protein Assay Kit, DAPI, HRP-conjugated anti-His Tag mAb and AEC Peroxidase Substrate Kit were purchased from Sorlarbio Life Sciences (Beijing, China). African swine fever virus positive sera were purchased from the National Center for Veterinary Culture Collection of China (Beijing, China).

### 2.2. Bioinformatical Analysis

The transmembrane domain of p54 was predicted by the online service TMHMM 2.0 (https://services.healthtech.dtu.dk/services/TMHMM-2.0/, accessed on 6 March 2025). B cell epitopes of p54 were predicted via different bioinformatic algorithms and tools including Kolaskar–Tongaonkar, Parker, Chou–Fasman, Karplus–Schulz, Emini, and BepiPred in the IEDB database (http://tools.iedb.org/bcell/, accessed on 13 March 2025). The three-dimensional structure of p54 was predicted by I-TASSER de novo modeling (https://zhanglab.ccmb.med.umich.edu, accessed on 27 March 2025) and SWISS-MODEL homologous modeling. The identified B cell epitopes were visualized on the model using PyMOL software (Version 3.1.3.1).

### 2.3. Expression and Purification of the Recombinant p54 Protein

The gene of the p54 protein was optimized for *E. coli* codon bias and obtained from NCBI (GenBank number OQ504956.1, isolate Pig/Jiangsu/LG/2021) [13]. The optimized p54 gene was synthesized by Sangon Biotech Co., Ltd. We designed a pair of primers for RT-PCR: the forward sequences of the primers were 5′-*CGCGGATCCATGGATTCTGAGTTCTTTCAGCCTG*-3′ and the reverse sequences of the primers were 5′-*CCGCTCGAGTCACAGAGAGTTCTCCAGATCCTTG*-3′. The p54 gene was inserted into pET-28a using a double digestion, amplification, and ligation process, and was named pET-28a-p54. The recombinant plasmid was transformed into BL21 receptor cells, which were cultured in a shaker at 37 °C for 4 h, until OD600 nm = 0.6. Then, 0.2 mmol/L IPTG was added and the expression was induced at 16 °C, 180 r/min for 10 h. The bacterium was enriched, and the supernatant and precipitate were separated using centrifugation at 4 °C and 12,000 r/min for 15 min. We purified the broken supernatant with reference to the Ni-NTA Agarose Operation Technical Manual. The concentration of p54 protein was determined using the BCA method, and it was quantified as 1 mg/mL and then dispensed and stored at −80 °C for backup. The proteins were characterized by Western blot with His-tagged antibody diluted at a ratio of 1:1000 as the primary antibody. HRP-labeled rabbit anti-mouse IgG antibody diluted at a ratio of 1:5000 was used as the secondary antibody, and the color was developed and analyzed using Western blotting.

### 2.4. Mouse Immunization and Generation of Monoclonal Antibodies

Five 6–8 weeks-old female BALB/c mice (recorded as mice 1 to 5, respectively) were obtained from the Laboratory Animal Centre of Zhengzhou University (Zhengzhou, China). Purified recombinant p54 protein was emulsified by mixing 1:1 with Fuchs’ complete adjuvant. The mice were immunized using subcutaneous multipoint injection at a dose of 25 μg each. The mice with the highest antibody potency were selected for reimmunization 3 days (d) before fusion and each mouse was injected intraperitoneally with 50 μg of recombinant p54 protein. The mice were euthanized, the splenocytes were isolated, and cell fusion of the splenocytes with logarithmic growth phase SP2/0 cells was performed under the action of PEG1450. After 7 d of cell fusion, 100 μL of culture supernatant was taken and detected by indirect ELISA. Positive hybridoma cells were subcloned two to three times until the antibody positivity rate reached 100%. The hybridoma cell lines with stable antibody secretion were expanded and frozen in liquid nitrogen for long-term storage.

### 2.5. Western Blot

HEK293T cells were transfected with pET-28a and pET-28a-p54 plasmids, and intracellular proteins were collected for Western blot verification. pET-28a and pET-28a-p54 plasmids were diluted with HIS-tagged antibody or positive serum at a ratio of 1:1000 as the primary antibody. After incubation, the samples were washed three times in TBST for 10 min each time. Then, the samples were immunoblotted with HRP-labeled goat anti-mouse IgG diluted at a ratio of 1:5000 as the secondary antibody and incubated at room temperature for 1 h. Ultrasensitive ECL chemiluminescence developing reagent was added to develop the color and the results of immunoblotting were analyzed.

### 2.6. Indirect ELISA

Dilute the recombinant p54 protein to 2 μg/mL in 1× CBS and encapsulate the plate overnight at 4 °C. Discard the coating solution, add 100 μL TBST to each well, and wash 3 times; close the samples with 5% skimmed milk for 2 h at 37 °C. Discard the coating solution and add TBST for 3 washes, 100 μL/well, 10 min each time. Add the hybridoma cell culture supernatant to the enzyme plate as the primary antibody, setting up negative and positive control at the same time. Let the control react at 37 °C for 1 h and wash with TBST 3 times; add 100 μL of HRP-labeled goat anti-mouse IgG (1:2500), let it react at 37 °C for 1 h, and wash with TBST 3 times. Add 100 μL 3,3′,5,5′-tetramethylbenzidine (TMB) color development solution at 37 °C for 15 min. Terminate the reaction by adding 100 μL of 2 mol/L H_2_SO_4_. Determine the OD450 nm value of each well. Examine the OD450 nm value of the sample and measure the negative sample with an enzyme marker. Test the OD450 nm of the sample; a negative sample (S/N) > 2.1 is determined to be positive.

### 2.7. Immunofluorescence Assay (IFA)

IFA demonstrated anti-p54 mAb specificity. HEK293T cells were transfected with pET-28a-p54 plasmids for 24 h. The slides were fixed with 4 moL/L paraformaldehyde. Monoclonal cell culture supernatant was used as the primary antibody, HRP-labeled goat anti-mouse IgG, diluted at a ratio of 1:200, was used as the secondary antibody. After fixing the slides, the nuclei of the cells were stained simultaneously with DAPI (Solarbio, Beijing, China). Fluorescence microscopy (ZEISS, Jena, Germany) was used for visual fluorescence signals after the final wash.

### 2.8. Peptide Design and Synthesis

The protein of p54 (184 aa) was analyzed using bioinformatics. The Garnier–Robson, I-TASSER, and Chou–Fasman methods were used to predict the secondary structure of p54. The hydrophilicity of proteins was predicted according to the Kyte and Doolittle method, the antigenicity of proteins was predicted according to the Jameson–Wolf method, and the surface accessibility of proteins was predicted according to the Emini method. After analyzing the predicted data, the B cell antigenic epitope of p54 was estimated. The antigenicity index of the peptides was analyzed using Wu’s antigenicity index analysis. The whole ASFV p54 truncation library was generated by systematically truncating the discovered epitope peptides with an offset of six amino acids to improve the mapping resolution. Each peptide’s N terminus was cysteine for conjugation (Table 1).

### 2.9. Dot-Blot Assay and Peptide ELISA

A piece of NC membrane was taken and moistened with PBS, and then the NC membrane was naturally air-dried at room temperature. Then, 2 μL of the peptide was coupled with BSA to be tested—the positive control was the p54 protein and the negative control was the BSA—and a spot sample on the membrane was air-dried at room temperature. Next, sealing solution was used to seal the NC membrane overnight at 4C; after sealing, the primary antibody and secondary antibody were incubated sequentially. Finally, AEC substrate color development solution was prepared, the NC membrane was washed to ensure that the color development solution was evenly distributed on the membrane, and color development was carried out for 5 min. At the end of the color development, the NC membrane was soaked with deionized water to terminate the color development, and the color development results were photographed and recorded.

### 2.10. Alanine-Scanning Mutagenesis and Epitope Mapping of Anti-p54 MAbs

Alanine mutation identified P1 and P4’s essential amino acid locations. Peptides were swapped with alanine (A) one by one. Mutant peptides were first linked with BSA to make antigens. Glycine replaced alanine (A) in the sequence. P1 and P4 were the positive controls and BSA was the negative control on the 96-well ELISA plates with linked peptides. The primary and secondary antibodies were monoclonal antibodies and Goat Anti-Mouse IgG/HRP.

### 2.11. Homology Analysis and Structure Prediction of the Identified P54 Epitopes

The gene sequences (Fasta format) of different subtypes of ASFV p54 were downloaded from NCBI and the amino acid sequences of the dominant epitopes were analyzed for their conservation among different subtypes. Twenty-two genotypes of 51 typical ASFV strains were selected for precision analysis using Clustal W in MEGA X.0. and Jalview (version 2.11.1.4). The phylogenetic tree was generated using the MEGA-X software (version 10.2.6, Mega Limited, Auckland, New Zealand). The spatial 3D structure of the ASFV p54 protein was modeled via the I-TASSER online website. PyMOL structural sequence analysis mapping software ((Version 3.1.3.1).) was used to display the eight overlapping peptides on the 3D model of the HPV16 L1 protein according to the amino acid sequences corresponding to p1–p8, and to analyze the structural features of the peptides.

### 2.12. Statistical Analysis

ChemiDoc MP Imaging System was used to analyze the SDS-PAGE, Western blot, and dot blot. GraphPad Prism 8.0 software was used for all statistical analyses and data visualizations. This document was written using Microsoft Office Professional Plus Word 2019. This study’s figures were displayed using Microsoft Office Professional Plus PowerPoint 2019.

## 3. Results

### 3.1. Expression and Purification of p54 Protein

**Figure 1:**
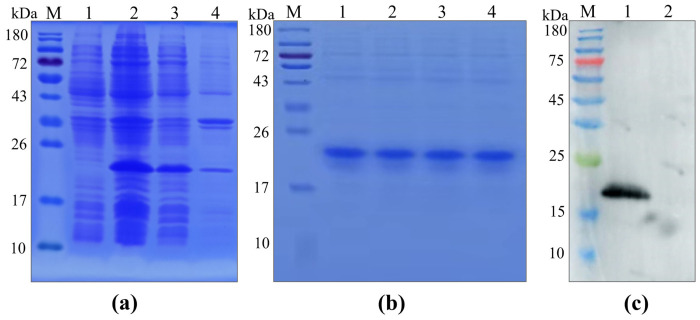
Expression, purification, and immunoreactivity of recombinant ASFV p54 protein. (**a**) SDS-PAGE analysis of p54 protein expression. Lane M: protein molecular weight marker; Lane 1: whole bacterial lysate before induction; Lane 2: whole bacterial lysate after IPTG induction; Lane 3: whole bacterial lysate after IPTG induction (replicate); Lane 4: soluble fraction (supernatant after ultrasonication). The arrow indicates the expressed p54 protein band at approximately 19 kDa. (**b**) Purification of p54 protein using Ni-NTA affinity chromatography. Lanes 1–4: elution fractions of recombinant p54 protein collected from the Ni-NTA column. The purified p54 protein appears as a single major band at 19 kDa. (**c**) Western blot identification of recombinant p54 protein using ASFV-positive pig serum. Lane M: protein marker; Lane 1: purified p54 protein probed with ASFV-positive serum; Lane 2: purified p54 protein probed with ASFV-negative serum (negative control).

To better fit the improved expression profile, codon use bias was altered. The relative codon frequency distribution is displayed in Figure S1A. Figure S1B illustrates the compatibility of the codon usage profile between the optimization sequence (shown in red) and host (shown in blue). The better the curve matches, the greater the compatibility. Unfavorable peaks were eliminated and the GC content was increased from 47.2 to 57.5% (Figure S1C). p54 was successfully expressed, according to the SDS-PAGE data (Figure 1a). p54 was highly pure after being refined using Ni-NTA affinity chromatography columns and it moved with an apparent molecular mass of roughly 19 kDa (Figure 1b). The purified p54 protein reacted with ASFV-positive serum during Western blotting (Figure 1c). The band size was consistent with the expected size of 19 kd.

### 3.2. P54 Immunization and Antibody Responses in Mice

The immunological process was followed while immunizing BLAB/C mice (Supplementary Figure S2A). Serum samples were gathered and ELISA was used to detect them. Compared with the negative control group, the serum antibody titer of the p54-immunized group was as high as 1:25,600, as shown in Supplementary Figure S2B. This finding suggests that the p54 vaccination generated a substantial antibody response in mice. Meanwhile, the outcome showed that the NO. 2 mouse had larger antibody titers than the NO. 1 and NO. 3 mice. As a result, the NO. 2 mouse was chosen for further mAbs preparation.

### 3.3. Identification and Characterization of Monoclonal Antibodies Against p54 Protein

**Figure 2:**
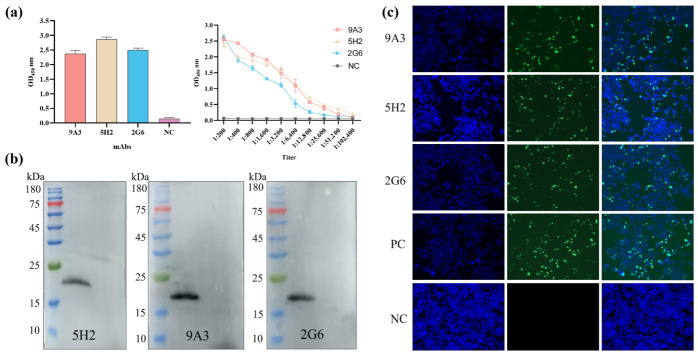
Characterization of mAbs. (**a**) Indirect ELISA was used to analyze the binding ability of three mAbs to p54 of ASFV. (**b**) Western blotting was used to analyze the reactivity of the mAbs with linearized protein p54. The total proteins of the *E. coli* BL21(DE3) cells were transformed with the recombinant plasmid pET28a-p54. The size of the target protein was estimated to be 19 kDa. (**c**) IFA was used to analyze the reactivity between the three mAbs and the ASFV p54 protein. The green color is anti-p54 mAbs; the blue color is the nucleus; the negative control (NC) was untransfected pLVX-p54 plasmid incubated with positive serum; the positive control (PC) was transfected pLVX-p54 plasmid incubated with positive serum (the positive serum was eyeball blood from mice that was used to screen for these monoclonal antibodies). Scale bars, 50 μm.

The cell clone expanded in the plate seven days after cell union and subsequently grew larger. Following a series of screenings, three mAb-secreting hybridoma cell lines generating the necessary antibodies were identified, namely 9A3, 5H2, and 2G6. In this work, the OD450 readings of serum from mice to day 56 were determined using indirect ELISA. The results showed that the highest potency of the p54 protein-encapsulated serum was 1:5.12 × 10^5^, whereas the control group did not produce any specific antibody (Figure 2a). The mAb subtype analysis revealed three IgG1 subclasses and all mAbs were of the Kappa light chain type (Table 2). The results of Western blotting experiments showed that all three p54 monoclonal antibody strains obtained reacted specifically with the p54 protein, which is about 19 KD (Figure 2b). Then, IFA results showed that all three monoclonal antibodies bound well to the intracellular ASFV p54 transient expression product and green fluorescence was visible under the microscope, localized in the cytoplasm. No fluorescence was presented in the empty vector transfection well control (Figure 2c).

### 3.4. Epitope Mapping of Anti-p54 MAbs

**Figure 3:**
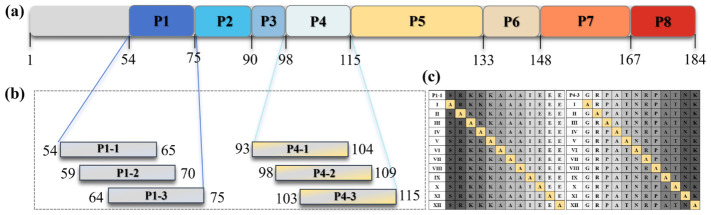
Schematic of p54 fragments used for epitope mapping. (**a**) A schematic of truncated fragments for primary epitope mapping; (**b**) a schematic of truncated fragments for P1 and P4 precise epitope mapping. Blue: P1 peptide; light blue: P4 peptide.; (**c**) a schematic of truncated fragments, and the alanine scan for P1-1 and P4-3 precise epitope mapping. Red indicates alanine residues; gray indicates normal amino acid residues (yellow: alanine mutation sites).

**Figure 4:**
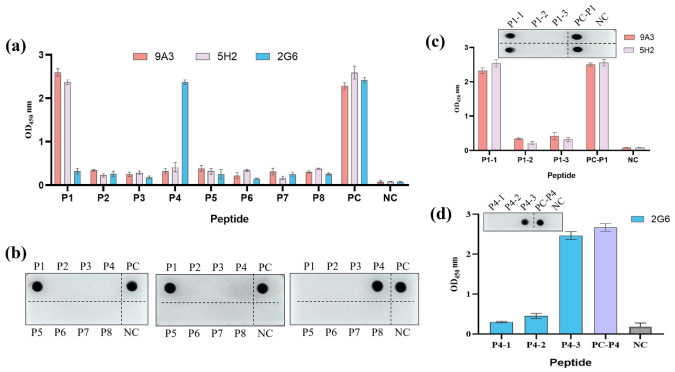
Map of the linear B cell epitopes of the p54 protein using the peptide ELISA and dot-blot assay. (**a**) Binding of mAbs with the overlapped peptides covering the p54 protein was determined using peptide ELISA. Positive control (PC), p54. Negative control (NC), BSA. (**b**) Binding of mAbs with the overlapped peptides covering the p54 protein was determined using dot-blot ELISA. PC, p54. NC, BSA. (**c**) Binding of mAbs with the truncated peptides derived from P1 was detected using dot-blot ELISA and peptide ELISA. (**d**) Binding of mAbs with the truncated peptides derived from P4 was detected using dot-blot ELISA and peptide ELISA (PC-P4, positive control).

To map the B cell epitopes of p54 recognized by these mAbs for the first time, the intracellular region of the ASFV p54 protein was overlappingly truncated into eight segments using peptide scanning (the overlapping peptide method), with five amino acid residues overlapping between each short peptide segment. A schematic of the synthetic peptides is shown in Figure 3a. Validation of the preliminary screening of B cell epitopes in the intracellular region of p54 using indirect ELISA is shown in Figure 4a and using Dot-ELISA in Figure 4b. The results showed that P1 (54–75) was recognized by mAbs 9A3 and 5H2, while P4 (93–115) was recognized by mAb 2G6. The p54 protein also reacted well with the monoclonal antibody, while the remaining short peptides did not react with the monoclonal antibody. Peptides P1 and P4 were further truncated to finely localize the B cell epitope of p54 (Figure 3b). The experimental results showed that monoclonal antibodies 9A3 and 5H2 recognized the P1-1 peptide, and monoclonal antibody 2G6 recognized the P4-3 polypeptide. Therefore, the epitopes were localized to 54SRKKKAAAIEE65 and 104GRPATNRPATNK115 (Figure 4c,d).

### 3.5. Identification of the Critical Amino Acid Residues of the Precise B Cell Epitopes

**Figure 5:**
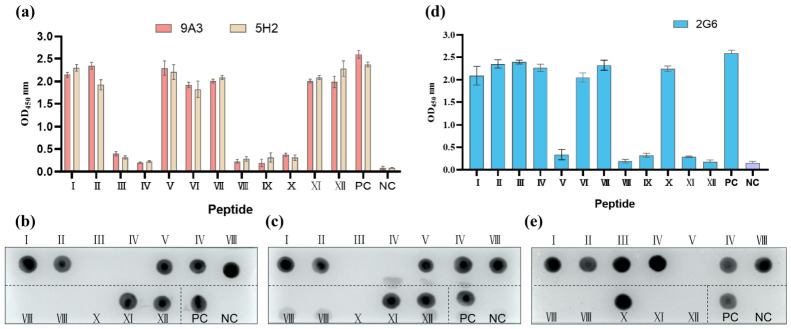
Alanine-scanning mutation was used to identify the key amino acid sites in mAbs binding. (**a**) The binding ability of mutation sequences of peptide P1-1 to mAbs 9A3 and 5H2. (**b**) The binding ability of mutation sequences of peptide P4-3 to mAbs 2G6. (**c**) Binding of mAbs 9A3 with the overlapped peptides covering the P1-1 protein was determined using dot-blot ELISA. PC, P1. NC, BSA. (**d**) Binding of mAbs 5H2 with the overlapped peptides covering the P1-1 protein was determined using dot-blot ELISA. PC, P1. NC, BSA. (**e**) Binding of mAbs 2G6 with the overlapped peptides covering the P4-3 protein was determined using dot-blot ELISA. PC, P4. NC, BSA.

Stepwise sequential alanine mutation scans were performed at the N- or C-terminus of the P1 and P4 polypeptide sequences to better define more precise linear epitopes. The alanine scan of the synthetic peptides is shown in Figure 3c. The results showed that, when the 56K, 57K, 60A, 61A, and 62I residues were substituted with the alanine/glycine, the peptide of P1-1 did not bind with the two mAbs 9A3 and 5H2 (Figure 5a). The results of Dot-ELISA were consistent with the results of the ELISA (Figure 5b,c). In the same way, Figure 5d,e shows that the residues 108T, 110R, 111P, 113T, and 114N played a key role in the binding of P4-3 to mAbs 2G6. Therefore, the minimal linear epitope recognized by 9A3 and 5H2 was ^56^KKKAAAI^62^ and the minimal linear epitope recognized by 2G6 was ^108^TNRPATN^114^.

### 3.6. The Conservation of the Identified Linear Epitope Among Different ASFV Reference Strains

**Figure 6:**
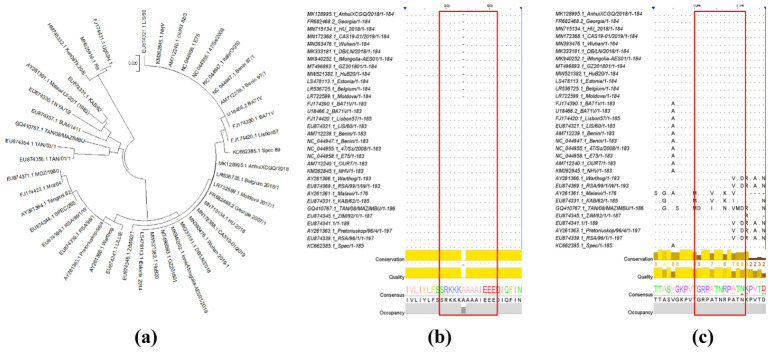
The conservation of the identified epitope among different ASFV reference strains. (**a**) Genotyping of 51 ASFV strains including 22 genotypes based on the p54 protein sequence. The phylogenetic tree was constructed using the neighbor-joining method in MEGA X. (**b**,**c**) Alignment of p54 protein sequence from different ASFV strains. The p54 sequences were aligned using Jalview software. Matching residues are denoted with black dots, while gap regions are denoted with dashed lines; the coordinate of the amino acid in the alignment is specified on the top and right terminus for each sequence. Epitope regions of 56–62 aa recognized by 9A3 and 5H2, and 108–114 aa recognized by 2G6 are circled with red.

In order to evaluate whether these epitope regions were consistent across ASFV genotypes, we analyzed the p54 sequences of 51 ASFV isolates (including 22 genotypes). Phylogenetic analysis is shown in Figure 6a; the amino acids of p54 among different ASFV strains were highly conserved. In particular, the 56KKKAAAI62 region of p54 was completely conserved among different ASFVs (Figure 6b). As can be seen from Figure 6c, the identified peptide in the ^108^TNRPATN^114^ region was comparatively conserved (up to 90% identity) but less conserved with individual mutations in the three strains AY261361.1, EU874331.1, and GQ410767.1.

### 3.7. Structural Analysis of the Identified Linear B Cell Epitopes

**Figure 7:**
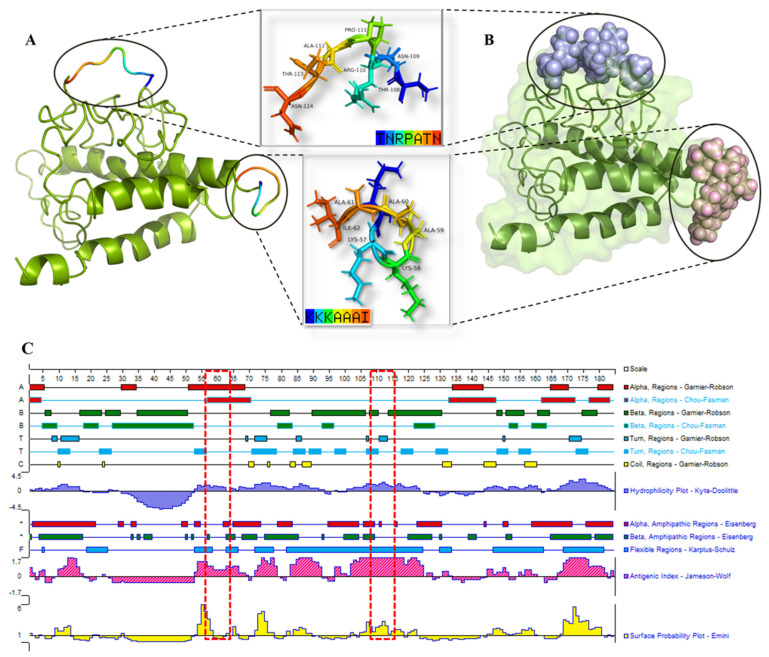
Localization of identified mAb-binding epitopes on the predicted 3D structure of the extracellular domain of the ASFV p54 protein. (**A**) The 3D model of p54 and the spatial position of epitopes ^56^KKKAAAI^62^ and ^108^TNRPATN^114^ are shown in the picture (the epitope amino acids in rainbow color; other amino acids in green color); (**B**) the p54 protein structure exhibits surface pattern in light green, with relative locations of the mAb-binding epitope ^56^KKKAAAI^62^ (recognized by mAb 9A3 and 5H2) marked in light pink and the location of the mAb-binding epitope ^108^TNRPATN^114^ (recognized by mAbs 2G6) marked in light blue; (**C**) secondary structural prediction of the core epitope by DNASTAR software. ^56^KKKAAAI^62^ and ^108^TNRPATN^114^ are indicated in red.

We obtained a more accurate structural model of the ASFV p54 protein with homology modeling using Phyre2. The 3D homology modeling structure is shown by both cartoons (Figure 7A) and spheres (Figure 7B). The spatial structure was drawn using PyMOL software, showing the position of the novel linear B cell epitope 56KKKAAAI62 (recognized by 9A3 and 5H2, marked in light pink) and 108TNRPATN114 (recognized by 2G6, marked in light blue).

The secondary structure properties of the merged sequences were analyzed using the Protean panel in the DNASTAR Lasergene 18.0.3 as shown in Figure 7C. There are folded, helical, and corner structures in the ^56^KKKAAAI^62^ peptide. The β-folded structure is present in the 108TNRPATN114 peptide. Therefore, in combining the experimental results and predictions, ^56^KKKAAAI^62^ and ^108^TNRPATN^114^ were the possible B cell epitope regions of p54.

### 3.8. Identification of Epitope-Specific Peptides

**Figure 8:**
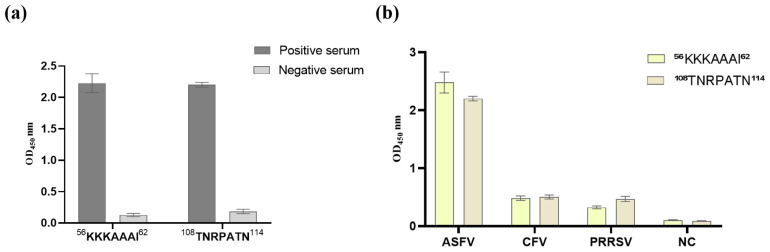
Reactivity of the Pi5 epitope with ASFV-positive sera. (**a**) Indirect ELISA detection of ASFV antibodies in pig sera using the two epitope peptides (^56^KKKAAAI^62^ and ^108^TNRPATN^114^) as coating antigens (2 µg/mL). ASFV-positive (*n* = 3) and -negative (*n* = 3) pig sera were diluted at 1:1000. OD450 values are shown as mean ± SD. (**b**) Specificity analysis. Sera positive for ASFV, classical swine fever virus (CSFV), and porcine reproductive and respiratory syndrome virus (PRRSV) were tested under the same conditions. Only ASFV-positive sera showed strong reactivity, confirming the high specificity of both epitopes.

To evaluate whether the identified epitopes are immunogenic in pigs, we performed indirect ELISAs using synthetic peptides corresponding to ^56^KKKAAA^62^ and ^108^TNRPATN^114^ as coating antigens. As shown in Figure 8a, both peptides strongly reacted with ASFV-positive pig sera (diluted 1:1000) but not with negative sera, indicating that these epitopes are recognized by antibodies generated during ASFV infection in pigs. Furthermore, specificity testing revealed that neither peptide cross-reacted with CSFV- or PRRSV-positive sera (Figure 8b), demonstrating excellent specificity. These results confirm the immunogenicity of the two epitopes in the natural host and support their potential utility as serological diagnostic markers for ASF.

## 4. Discussion

African swine fever (ASF) is an acute hemorrhagic disease with a case fatality rate approaching 100%, posing a severe threat to the global swine industry [14]. In the absence of effective vaccines, understanding the molecular basis of antigen–antibody interactions, particularly the precise mapping of immunodominant epitopes, is critical for the development of subunit vaccines and diagnostic tools [15]. Among the various structural proteins of ASFV, the early-expressed p54 protein is highly immunogenic and induces a persistent antibody response during infection, making it a valuable target for serological diagnosis and vaccine development [16,17]. Monoclonal antibodies (mAbs) have been widely used as key reagents for virus detection and previous studies have employed mAbs against p54 to detect ASFV infection in peripheral blood. In recent years, several groups have mapped linear B cell epitopes on p54, identifying multiple immunodominant regions within the N-terminus (1–20aa [18], 5–9aa [19], 15–35aa, aa60–72 [12], 60–79aa [20,21], 66–76aa [22], 76–81aa [23]) and C-terminus (106–132aa [18], 117–128aa [21], 128–148aa [12], 143–152aa [11], 162–175aa [12], 175–184aa [24]). Some of these epitopes are highly conserved across different genotypes and show diagnostic potential; others have been shown to mimic conformational epitopes or dominate early antibody responses. However, a systematic, high-resolution scan of the full-length p54 protein has not been performed and functional validation of the identified epitopes remains limited. Notably, subsequent studies have demonstrated that p54 alone is ineffective as a subunit vaccine [25,26], suggesting that the application of p54 epitopes should shift toward improving diagnostic tools and rationally designing multi-epitope vaccines rather than focusing solely on standalone vaccine development.

In this study, we expressed and purified a truncated form of the p54 protein (aa 54–184) in *E. coli*, obtaining highly pure antigen (>90%) for efficient mAb screening. Three p54-specific mAbs (9A3, 5H2, and 2G6) were generated. Using a combination of bioinformatics prediction and overlapping peptide scanning, we identified two linear B cell epitope regions: ^54^SRKKKAAAIEEEDIQFINPYQD^75^ and ^93^ATTASVGKPVTGRPATNRPATNK^115^. Further fine mapping and alanine-scanning mutagenesis defined the minimal linear epitopes as ^56^KKKAAAI^62^ (recognized by 9A3 and 5H2) and ^108^TNRPATN^114^ (recognized by 2G6). Of these, ^56^KKKAAAI^62^ is reported as a novel linear epitope for the first time. The alanine-scanning results showed that residues 56K, 57K, 60A, 61A, and 62I, as well as 108T, 110R, 111P, 113T, and 114N, are critical for antibody binding. These core residues provide precise molecular targets for subsequent epitope modification and functional optimization.

In this study, we used a complete overlapping peptide library covering the intracellular region of p54 (aa 54–184), consisting of eight peptides, and validated the results using both dot-blot and peptide ELISA, thereby avoiding the omission of potential epitopes due to gaps in peptide design. Through stepwise truncation and alanine-scanning mutagenesis, we achieved high-resolution epitope mapping and identified the key amino acid residues, a level of detail rarely reported in previous p54 epitope studies. The newly identified epitope ^56^KKKAAAI^62^ is located in a region (near aa 55–65) that has received little attention, thereby expanding the antigenic epitope map of p54. Moreover, the fact that two mAbs (9A3 and 5H2) recognize the same epitope suggests that this region is immunodominant and may serve as an ideal target for diagnostic reagent development. In a previous study, Zhang et al. [9] reported three linear B cell epitopes on p54 (^60^AAIEEEDIQFINP^72^, ^128^MATGGPAAAPAAASAPAHPAE^148^, and ^163^MSAIENLRQRNTY^175^) using three mAbs (6B11, 3E3, and 3C10). Their study focused on diagnostic ELISA development. In contrast, our study identified two different epitopes (^56^KKKAAAI^62^ and ^108^TNRPATN^114^) using independent mAb panels, and we further performed alanine-scanning mutagenesis to define the critical residues required for antibody binding. Importantly, the epitope ^56^KKKAAAI^62^ has not been reported previously and represents a truly novel linear B cell epitope. Moreover, our conservation analysis covered 22 ASFV genotypes, demonstrating that this novel epitope is 100% conserved across all major genotypes. Thus, our work complements and extends the findings of Zhang et al. by providing higher-resolution epitope information and a broader genotype coverage.

Sequence conservation analysis is essential for evaluating the practical applicability of epitopes. We selected 51 representative ASFV strains covering 22 genotypes and analyzed the conservation of the two identified epitopes. The results showed that ^56^KKKAAAI^62^ is completely conserved (100%) among all analyzed strains, while ^108^TNRPATN^114^ exhibited >90% conservation, with only occasional amino acid substitutions in a few strains (e.g., AY261361.1, EU874331.1, and GQ410767.1). This high degree of conservation suggests that these two epitopes and their corresponding antibodies have the potential for broad-spectrum detection of ASFV infections caused by different genotypes, particularly for monitoring the predominant genotypes I and II circulating in China and neighboring countries.

We further predicted the three-dimensional structure of p54 using homology modeling and mapped the two epitopes onto the structural model. The ^56^KKKAAAI^62^ region displays a mixed structure comprising folds, helices, and turns, whereas the ^108^TNRPATN^114^ region is predominantly β-sheet. These structural features are consistent with the notion that linear epitopes are often located on the surface or in the flexible regions of proteins, further supporting our experimental localization. It should be noted that, because the crystal structure of full-length p54 has not been resolved, the resolution of our homology model is limited. Future validation of the spatial conformation of these epitopes using site-directed mutagenesis or structural biology approaches (e.g., X-ray crystallography, cryo-EM) is warranted.

The findings of this study have clear potential applications. The highly conserved linear epitopes identified can serve as antigenic markers for the development of epitope-based ELISA methods for the serological diagnosis of ASFV infection. They can also be combined with epitopes from other proteins (e.g., p30, p72) to construct multi-epitope diagnostic antigens, thereby improving assay sensitivity and specificity. Although p54 alone is ineffective as a subunit vaccine, multi-epitope chimeric vaccines or peptide-based vaccines containing ^56^KKKAAAI^62^ and ^108^TNRPATN^114^ may still induce partial protective immunity through specific antibody responses, a possibility that warrants further evaluation in animal models. Additionally, the mAbs generated in this study could be used to develop competitive ELISA kits for DIVA (differentiation of infected from vaccinated animals) strategies.

## 5. Conclusions

In summary, this study successfully screened and finely mapped two linear B cell epitopes on the ASFV p54 protein, among which ^56^KKKAAAI^62^ is reported as a novel epitope for the first time. Both epitopes are highly conserved across different ASFV genotypes and the key amino acid residues involved in antibody recognition have been clearly defined. These results provide an important theoretical basis and molecular targets for the development of epitope-based diagnostic reagents, multi-epitope vaccines, and therapeutic antibody drugs against ASFV. Future studies will focus on evaluating the diagnostic performance of these epitopes in clinical settings and exploring their application in peptide vaccine development.

## Figures and Tables

**Table 1:** Amino acid sequences of overlapping synthetic peptides of p54 protein.

Peptide ID	Sequence of Amino Acid	Position
P1	SRKKKAAAIEEEDIQFINPYQD	54–75
P2	INPYQDQQWVEVTPQPGTSKP	70–90
P3	PGTSKPAGATTASV	85–98
P4	ATTASVGKPVTGRPATNRPATNK	93–115
P5	RPATNRPATNKPVTDNPVTDRLVMATGGP	110–133
P6	MATGGPAAAPAAASAPAHPAE	128–148
P7	PAHPAEPYTTVTTQNTASQTMSAIE	143–167
P8	TMSAIENLRQRNTYTHKDLENSL	162–184
P1-1	SRKKKAAAIEEE	54–65
P1-2	AAAIEEEDIQFI	59–70
P1-3	EEDIQFINPYQD	64–75
P4-1	ATTASVGKPVTG	93–104
P4-2	VGKPVTGRPATN	98–109
P4-3	GRPATNRPATNK	104–115

**Table 2:** Identification for subtypes of monoclonal antibodies.

No	mAbs	Type		Affinity Constant (L/mol)
		Heavy Chain	Light Chain	
1	2G6	IgG1	Kappa	5.7 × 10^6^
2	5H2	IgG1	Kappa	4.9 × 10^6^
3	9A3	IgG1	Kappa	8.3 × 10^6^

## Data Availability

The original contributions presented in this study are included in the article and Supplementary Material. Further inquiries can be directed to the corresponding author.

## Supplementary Materials

The following supporting information can be downloaded at https://www.mdpi.com/article/10.3390/microorganisms14071404/s1. Figure S1: Codon optimization of nucleocapsid protein p54 of ASFV; Figure S2: Immunization strategies and antibody responses in mice.
