# Supplementary material for: Novel Linear B Cell Epitopes of ASFV p54: Screening and Fine-Scale Mapping
Source: Microorganisms. 2026 Jun 25;14(7):1404. doi: 10.3390/microorganisms14071404 (PMC13414447; doi:10.3390/microorganisms14071404)
Supplement: Supplementary file 1 [file microorganisms-14-01404-s001.zip › Supplementary Material-Novel Linear B Cell Epitopes of ASFV p54 Screening and Fine-Scale Mapping.pdf]

Novel Linear B Cell Epitopes of ASFV p54: Screening and Fine-Scale Mapping

Haili Wang [1], Wenying Yan [1]， Xiao Liu [1]， Yanwei Wang [1]， Shulei Li [1], Linyi Bai [1],

Xiaomin Li [1], Yaxin Guo [1], Aiping Wang[1,2,*]

1 Longhu Laboratory of Advanced Immunology, Zhengzhou 450046, China

2 Henan Provincial Key Laboratory of Immunological Biology, Zhengzhou 450001, China

* Correspondence: Aiping Wang, E-mail: pingaw@126.com*Corresponding author at: School of advanced agricultural sciences, Peking University, Beijing 100871, China.

E-mail address: wanghailiguai@163.com(H. Wang), pingaw@126.com (A. Wang)

Supplementary Material

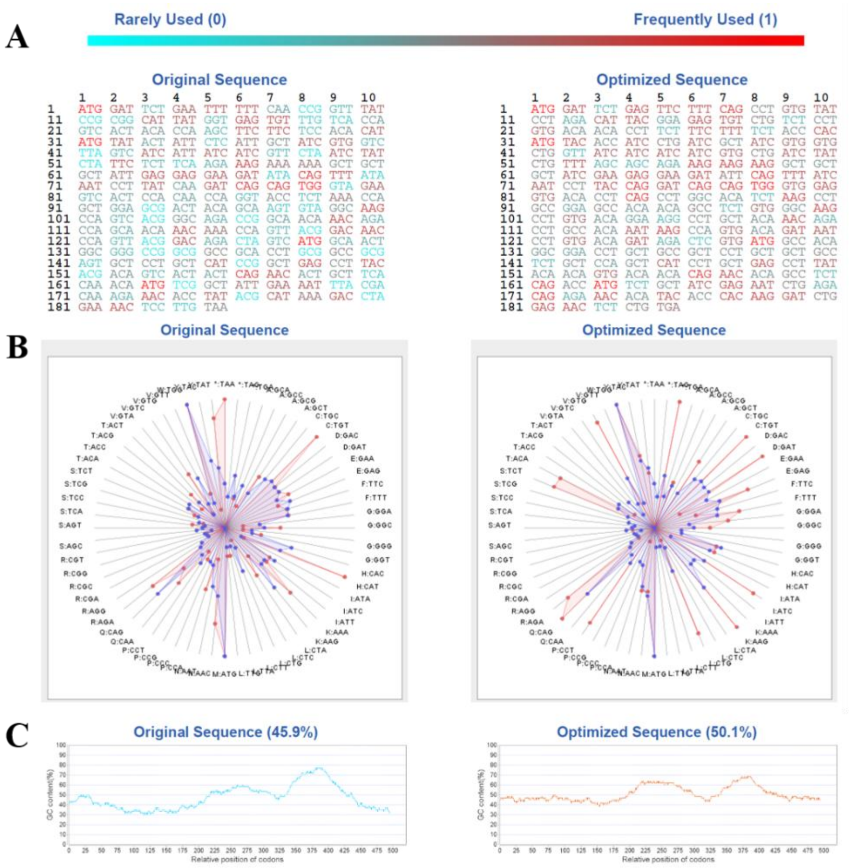

Fig. S1. Codon optimization of nucleocapsid protein p54 of ASFV.

A. Relative codon frequency distribution of p54 protein. Color of codons indicates the frequency for that codon, with respect to the host. Codons that are rarely used are shown in light blue color and frequently used are shown in red color. More red indicates the higher frequency was used and was better suited for the host codon bias, and resulting in higher expression.

B. Codon relative frequency radar plot. The relative codon frequency distribution show the frequency of each individual codons and this radar plot shows the suitability of the codon usage profile between optimized sequence (show in red) and host (shown in blue). A better curve match means it is more adequate.

C. GC content adjustment; The original GC content is 45.9%, and the optimized value is 50.1%

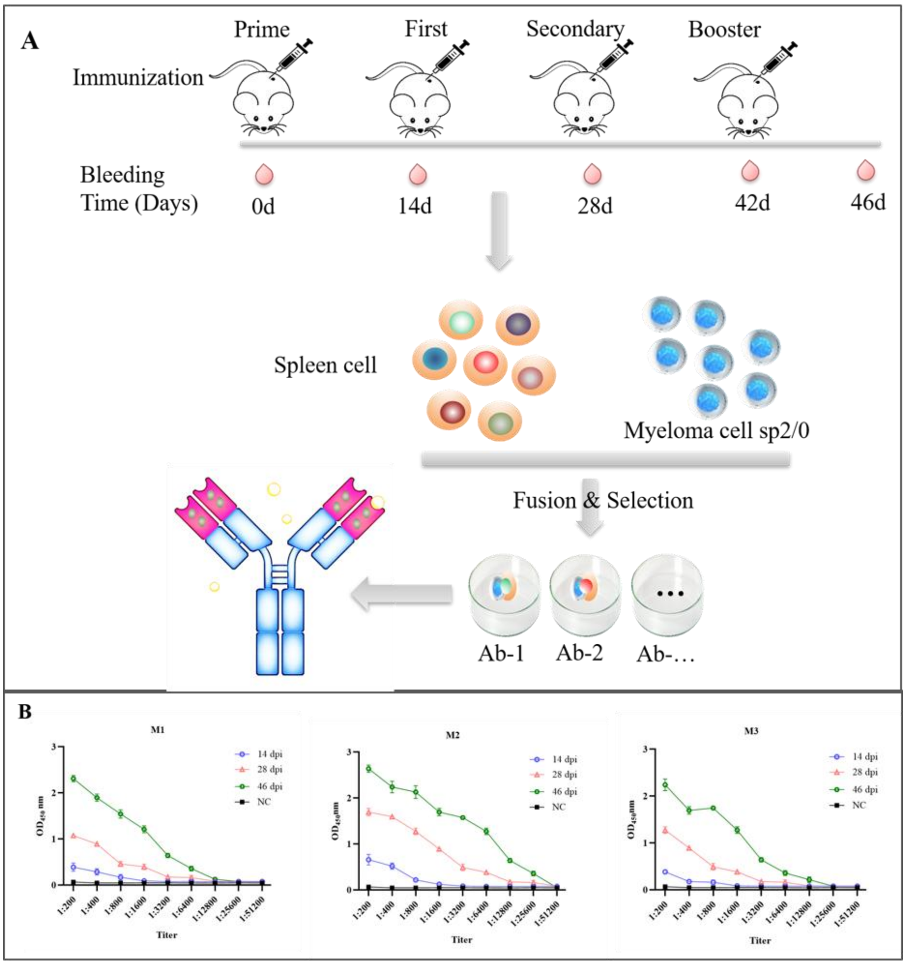

Fig. S2 Immunization strategies and antibody responses in mice.

A. Scheme of immunization and fusion. B cells and SP2/0 myeloma cells were fused by PEG1500 and selected in HAT medium. Finally, hybridoma cells producing the desired antibodies were screened by ELISA.

B. Titration of p54 antibodies in the mice sera after immunization carried out by ELISA. dpi, days post-prime immunizations. M1, M2 and M3 represent different mice.
